# Board of directors and earnings manipulation: evidence from regulatory change

**DOI:** 10.1186/s43093-022-00173-1

**Published:** 2022-12-19

**Authors:** Sattar Khan, Yasir Kamal, Muhammad Abbas, Shahid Hussain

**Affiliations:** 1 Institute of Management Sciences, Peshawar, Pakistan; 2 Department of Business Administration, Sarhad University of Science and Information Technology, Peshawar, Pakistan; 3 Department of Finance and Investment, NUST Business School (NBS), National University of Sciences and Technology (NUST), Islamabad, Pakistan

**Keywords:** Board attributes, Earnings management, Corporate governance, Board of directors, Code of corporate governance 2017, Pakistan

## Abstract

The purpose of this paper is to examine the effect of the board of directors’ related clauses such as independence, female director, CEO Duality and the expertise of director included in the Code of Corporate Governance 2017 (CCG-2017) on earnings management with the pre- and post-CCG-2017 analysis. This study has used the sample of 323 non-financial listed firms of the Pakistan Stock Exchange from 2015 to 2019. Data were manually collected from companies’ annual reports, and two proxies of earnings management have used: one is discretionary accruals and the other is real activity manipulation. The results of the study show that as compared to the pre-period of CCG-2017 in the post-period of CCG-2017 board independence, expertise and female inclusion has increased significantly. Moreover, board independence and financially expert directors are negatively related to discretionary accruals, while there is a positive relationship of female directors with discretionary accruals, which is also same for real activity manipulation. The findings also show that there is no relationship of board independence/outside directors and expert directors with real activity manipulation. This study recommended the CCG-2017 reforms introduced by the regulator. Moreover, we recommend that the regulator needs to augment the authentic independence of independent/outside directors in listed firms (concentrated ownership context) of Pakistan. This study adds its part in the corporate governance literature by focusing board attributes with regulatory reforms on earnings manipulation, which is lacking in the related literature in general and in Pakistan an emerging economy in particular.

## Introduction

The main objective of this study is to investigate the role of board of directors’ characteristics in restraining earnings management (hereafter, EM) and by checking the impact of Code of Corporate Governance 2017 (hereafter, CCG-2017) regulations and revisions in the board of directors’ related clauses on EM in Pakistan. According to Kao and Chen [35], most of the alleged EM practices in the USA, which was made in the late 1990s and early 2000s, was due to the inadequate monitoring of board members, which resulted the well-known accounting scandals such as Enron and WorldCom etc. Moreover, these accounting scandals were due to the EM practices by overstating sales and revenues. It was due to these scandals that American Congress introduced the famous Sarbanes–Oxley Act (hereafter, SOX) in 2002 in order to strengthen boards and audit committees to improve the financial reporting quality [19]. In order to improve the board oversight and cope with international trends the Securities and Exchange Commission of Pakistan (hereafter, SECP) implemented the CCG-2017^1^ to strengthen the board functions by including mandatory clauses of female, independent and expert directors in the boardrooms of the Pakistani listed firms. Moreover, the board of directors is the highest decision making and supervisory body in the companies’ management hierarchy, concerned literature documented two views about the functioning of board of directors in corporations, namely the resource dependence view and the agency view [1]. The former states that boards perform the monitoring role over self-interested managers [31]; this role will be performed by inclusion of more independent directors in the boards. The latter view assumes that boards performed the advisory and consoling roles in the functioning of corporations [56]. Moreover, diverse boards are come under resources dependence view which improves the functioning of the organizations. Aggarwal et al. [1] added that following the above views, the independent and diverse boards are possibly had superior monitoring which resulting improved financial performance in the organization. In addition to this, the composition of the board is one of the most important features of any corporate governance system. The study of Saona, Muro, and Alvarado [63] posits that to increase the monitoring and effectiveness of the boards the inclusion of external and independent directors is pertinent.

Although the board of directors (hereafter, BOD) has a very important role in corporate governance and in protecting the shareholder rights’ but there is considerable empirical evidence showing management involvement in earnings manipulating practices [58, 64, 78]. This evidence is based on developed countries. Therefore, the main motive of this research study is to check that whether BODs are involved in EM practices in Pakistan: a developing country or BOD is instrumental in constraining EM. Shaikh, Fei, Shaique, and Nazir [66] added that Pakistani firms’ ownership structure is unique in the sense that it is enormously concentrated; the reason behind this phenomenon is family ownership. In addition to this, the study of Hussain and Safdar [27] depicted that in Pakistan most of the firms have concentrated ownership and controlled by business families. Due to family orientation and ownership concentration in Pakistani listed firms, the majority of boards are consisted of family members and relatives. Therefore, focus has been made by the regulator (SECP) in the corporate governance codes, especially CCG-2017 to include the increased number of outside directors in boardrooms in Pakistani listed firms to ensure transparency and effective board monitoring for minority shareholder’s rights protection. Keeping in view the importance of independent, female and financially expert directors, this study is intending to check the role of the independent, financially expert as well as female directors in attenuating EM for the pre- and post-CCG-2017 period. In addition to this, in Pakistan researcher has linked ownership structure [, audit committee attributes [34, 40, 53], the board of directors attribute [36] and overall CG variables with EM [3, 28, 29, 44, 45]. To the best of our knowledge, there is no study in the concerned literature by linking board attributes to EM with focusing the impact of CCG-2017 in Pakistan.

Therefore, this study is intending to fill this gap in the literature by checking the effect of BOD-related reforms in CCG-2017 on EM. Moreover, in the international level BOD is linked with EM focusing on one EM proxy [6, 35, 50]]. However, this research study is focusing on two proxies of EM, such as discretionary accruals manipulation and real activity manipulation. Although the BOD and EM relationship is extensively researched in the international level, however, little evidence is there while focusing country-specific regulation such as CCG-2017. Therefore, this research study is intending to examine the BOD relationship with EM, while focusing the regulatory change in the CCG-2017 and its impact on EM in Pakistani context. Additionally, this research study will answer the research question that whether board attributes such as independence, expertise, CEO Duality and the female directorship, influencing earnings manipulation (both accrual and real activity manipulation) practices or not and CCG-2017 has any role in this regard.

In response to the study’s research objectives, questions and linking the country-specific CCG-2017 regulation with BOD on EM in Pakistan. The findings of the study reported that mean differences of the BODs’ attributes independence, board diversity, and board expertise are significantly different in the pre- and post-periods by 3, 2 and 2 percent. Furthermore, the regression results show that there is significant negative relationship of board independence and financially expert directors with EM in the post-CCG-2017 period and full-period regression, while in the pre-period there is no significant relationship between board independence and expert directors with EM. The study’s findings show that independent and financially expert directors are more effective in limiting EM practices in the post-CCG-2017 period as compared to the pre-CCG-2017 period. Moreover, the female proportion in the board is not related to EM in the post-CCG-2017 period as predicated; however, it is positively related to EM in full-period regression. These results show that study’s findings cannot support that gender diverse board in the post-CCG-2017 period is negatively related to EM.

As stated in the previous paragraphs, this study was aiming it to check the influence of CCG-2017 board-related clauses on EM, by doing so this research article contributed to the prevailing literature in the following ways. Firstly, this research study extended that line of research, which is related to EM and BODs. Furthermore, it has added its part to that aspect of corporate governance, which examines the BOD attributes influence of firms financial reporting quality. Secondly, this research study added in the corporate governance literature by focusing EM and government regulation in Pakistan, an emerging economy, while previous study was only focusing EM [15, 23, 36, 37, 47]. Furthermore, this paper extended the understanding of BOD attributes with EM, focusing Pakistan an emerging market. Thirdly, it is contributed to the literature on board independence and financially expert directors to be effective monitors in concentrated ownership structure where principal–principal conflict exists, while the female directorship is not rated as reported in related literature. On the other hand, previous studies have laid emphasize on one specific variable of board, such as board independence, board female diversity, board expertise or CEO attributes, while in this research study four board attributes have been taken. Lastly, to the best of our knowledge, this study investigated board attributes with EM focusing government regulation and taking into consideration of real activity manipulation, while the related literature only focused on discretionary accruals manipulation [35, 50, 71].

At last, the rest of the study has adopted the following scheme. The study’s context is given in the section "The context of Pakistan and different corporate governance regulations" in the "Related literature and hypotheses development" section, the theoretical framework, relevant literature and study’s hypotheses are given. The EM model, sample size details and the econometrics model of the study are given in "Methodology" section.  The "Empirical results" gives descriptive, correlation and regression analysis details with pre- and post-estimation tests, and discussion on results is also given. Lastly, this study ends with a "Conclusion".

## The context of Pakistan and different corporate governance regulations

The corporate sector of Pakistan consists of financial and non-financial institutions. These institutions are regulated by different regulators such as State Bank of Pakistan (SBP), SECP and (Pakistan Stock Exchange (PSX)). Financial institutions such as commercial banks, microfinance banks and development finance institutions are additionally regulated by SBP, while all non-financial institutions are regulated by SECP with a listing requirement in PSX. The first code of corporate governance (CCG) was implemented in 2002 in Pakistan. The 2002 CCG was based and formulated on the examples of different codes throughout the world [26]. In this code, it was encouraged to have an independent director in the listed companies, it made compulsory to have training programs for directors and have guidelines for establishment of an audit committee and Chief Executive Officer (henceforth, CEO), and chairman could be the same persons. The 2002 CCG have no clause of female and expert directors in the board of directors or board’s committees. After implementation of the 2002-CCG, many companies were de-listed from the stock exchanges due to non-compliance and lack of understanding of the code in the corporate sector in Pakistan [75]. After a decade of implementation, the 2002-CCG was revised in 2012 by implementing more demanding clauses regarding board of directors, board committees and compliance clauses.

In the CCG-2012, it is made mandatory that there must be at least one independent directors in the board, while in the CCG-2002 it is suggested to be one in the board [39]. In addition to this, it is preferred in the CCG-2012 to have 1/3 of independent directors in the board. Moreover, the independence of the directors in the board is further elaborated in the CCG-2012 as compared to CCG-2002 . The independence of Audit Committee was introduced in the CCG-2012, which was not given in the CCG-2002. The 2012 code does not include any clause of gender diversity and board expertise, while on the other hand in addition to audit committee, the Remuneration and Human Resource Committee were introduced in the CCG-2012 and made this code is a listing requirements in the PSX.

Due to some limitations such as lack of board diversity and expertise and in order to cope with international trends and practices, SECP revised the CCG-2012 at the end of 2017. It includes the mandatory inclusion of female director in the board and expertise members in the audit committee as well as reduced the multiple directorship from 7 to 5 companies. In addition to this, it reduced the executive directors to 1/3 of the board of directors; furthermore, it improves the board independence by including the clause ‘not less than two members or one-third of the total members’ should be independent in the board. Therefore, this research study is intended to check the impact of the recent past CCG-2017 along with BOD attributes on EM practices in Pakistan.

## Related literature and hypotheses development

### Theoretical framework of the study

According to the monitoring hypothesis of the agency theory, the shareholders appoint the BOD to keep watch on the management’s opportunistic behavior. Moreover, Jensen and Meckling [31] identified the conflict between shareholders (principals) and management (agents) and posited that BOD’s role is to monitor the management and protect the interests of all the shareholders. They further added that the monitoring function of the board eases the agency problem between the shareholders and management. Furthermore, the information asymmetry between the management and shareholders encourages the management to manipulate earnings for their vested interests, which is a deterrent to shareholders. In such a situation, the BOD, as an internal corporate governance mechanism, plays an essential role in preventing the management’s opportunistic behavior. In the same vein, the monitoring function of the board will be more effective when there are independent directors [16] and female directors, because female directors are more risk-averse, conservative, and ethically sensitive [80]. This research study’s hypotheses are formulated based on agency theory, which is consistent with the studies of [51, 60, 61, 80].

### Board independence and earnings manipulation

Independence in the board structure is one of the most essential elements. In every corporate governance code or law, independence is considered a vital part of that code or law and it is observed throughout the world. In addition to this, CG codes globally have an explicit ratio of independent directors in the board structure. Aguilera and Cuervo-Cazurra [2] added that 196 codes of 46 different countries for the period of 1978 to 2008 have explicit or implicit recommendations of having a balance ratio of independent (non-executive) and executive directors. In Pakistan due to concentrated ownership and family control, the board independence issue was not in the spotlight in the first two codes of CG. While in the recent CCG-2017, the issue of independence has been taken into account by the regulator and now it is mandatory for every listed company to have a minimum of two independent directors or one-third of total directors, whichever is higher. Independent directors in BOD are regarded better for supervision and monitoring purposes through which opportunistic behavior can be prevented. According to agency theory, the independence of directors in the boardroom is one of the most important features, which reduces the cost of the agency [63] and outside (independent) directors are unbiased in their decision making. They have lack of personal interest in the company and have no family relation within a company, so the higher the ratio, the less likely there will be earnings manipulation. Moreover, Chouaibi et al. [11] and Daghsni et al. [12] argued that board effectiveness is largely determined by independent and outside directors who are instrumental in enhancing control over management, minimizing their discretion on earnings, and reducing their opportunistic behaviors.

The literature relating to independent directors and EM is inconclusive. Alden, Al, Sukoharsono, and Andayani [4] added that board independence is the strongest CG indicator through which earnings manipulation can be mitigated. In addition to this, Yung-chuan Lee [79] argued that independent directors improve the quality of reported earnings and have a positive impact on the company’s reported earnings quality. Cheng, Chen, and Wang [10] added that independent directors reduce EM in situations where acquisition of company-related information is easy and cost-effective. In addition to this, Young et al., [78] posited that those board that have more independent and professional directors have a negative impact on earnings manipulation, which result in better board governance and monitoring. The literature review study of Man and Wong [46] indicated that BOD independence in the board improves the board’s (management) ability to monitor and control management’s EM practices. In addition to the above discussion, the studies of [9, 22, 54] provided evidence that independent-cum-outside directors are not linked to reduce EM, because only independence is not sufficient to keep watch on management, but it requires specific firm-related knowledge as well. In the case of Pakistan, where the dominance of family businesses and concentration of ownership are prevalent and the regulator encouraged the independence in the previous corporate codes (2002 and 2012) but did not make it mandatory. So there is a very low ratio of independent directors in corporate boards in Pakistan. The studies of [57, 65, 81] investigated CG and related variables with EM and found that board independence has no role in reducing EM in Pakistan. On the other hand, the results of the studies [59, 72] show that board independence is negatively related to EM. Based upon the above discussion and considering the mandatory inclusion of independent directors on the board, this research study will propose the following hypotheses,

#### H1:

In Pakistani non-financial listed firms, board independence is more effective in limiting earnings management (accrual and real EM) in the post-CCG-2017 period than in the pre-CCG-2017 period.

### Gender diversity in the board of directors and earnings manipulation

Diversity on the board is an important element for monitoring and controlling corporations. According to Man and Wong [46], gender diversity matters in EM because women in BODs give motivation, they have high moral principles, conservative in following EM strategies and more risk-averse towards EM practices. Moreover, Lakhal [43] added that diversity in BODs and the company’s top management lead to mitigation in EM. Wahid [74] posits that gender diversity on boards engages in less fraud and commits fewer mistakes in preparing and presenting financial statements. Moreover, Fernández-Temprano and Tejerina-Gaite [17] investigated that gender diversity in the board increases firm accounting performance, which means that having a female in the BOD reduces EM practices. Gul, Srinidhi, and Ng [21] documented that gender diversity on the board could improve the discussion quality of the board as well as improve the BOD oversight of the firm’s disclosures and financial reporting. In addition to this, studies such as [25, 52, 68] showed that there is no negative relationship between EM and gender diversity in the BOD. In the Pakistani context, the study of [72] shows that diversity in corporate boards in Pakistan reduces EM. Furthermore, they add that female directors are very viable in board rooms and their presence on boards strengthens the effectiveness of the BOD, which curtails EM. To sum up the above discussion, there are mixed results for gender diversity and EM in the relevant literature. A gender diverse board is considered a balanced board, so due to the importance of diversity (gender) in BOD, it is made mandatory for the listed companies in Pakistan to have one female director on the board. Because the inclusion of female directors on the CCG-2017 BOD may improve board quality, we expect that the post-period of CCG-2017 will have a negative impact on EM when compared to the pre-period of CCG-2017, so we formulate the study's second hypothesis as follows:

#### H2:

Gender diversity on the board of directors is more effective in limiting earnings management (accrual and real EM) in Pakistani non-financial listed firms in the post-CCG-2017 period than in the pre-CCG-2017 period.

### Financially expert directors and earnings manipulation

The term “financial literate (expert)” director means a person who has the membership of a SECP-recognized body of professional accountants or has a higher degree in finance from a Higher Education Commission (HEC) recognized university or equivalent institution (CCG-2017).^2^ It is widely believed that expertise in BOD is vital for reducing EM. According to Yeung and Lento [77], there are two perspectives on the board of directors’ role in companies: one is the agency theory perspective and the other is the resource dependence theory perceptive. The agency view asserts that financially expert directors in the company provides efficient governance, which effectively deals with uncertain situations. This quality of directors, especially accounting expertise, is one of the crucial features in their monitoring role [55]. This quality of directors constrains management from EM. As one of the most important functions of the BOD is to monitor the management, [67] added that the monitoring role of the BOD requires accounting knowledge, which is effective in controlling EM and making financial reports more transparent. Although the monitoring role is paramount for the directors, the advisory role is also the most important, which will be possible for them by having not only professional knowledge but also past working experience. Therefore, by having professional and past working experience, expert directors will reduce earnings manipulation. The study by Xie, Davidson and Dadalt [76] documented that financially expertise in BOD results in lower EM. On the contrary to this view, Ruparatne and Meegaswatte [24] added that expertise in BOD cannot be helpful to reduce EM. They further added that the financial expert director in BOD may use their intellectual skills and abilities to mask the accounting figure and indulge in EM practice. The study of [67] posited that for the monitoring process and to make the financial statements more transparent, directors must have expertise (accounting and past working experience) which will enable them to curb EM. Based on this argument and the scarcity of literature of board expertise and EM, this study will propose the following hypothesis,

#### H3:

Financially Expertise directors is more effective in limiting earnings management (accrual and real EM) in the post-CCG-2017 period as compared to pre-CCG-2017 in the Pakistani non-financial listed firms.

### CEO duality and earnings manipulation

The duality of the CEO in an organization means that one person holds the positions of CEO and chairman of the board. The CEO duality is one of the most important internal control mechanisms of corporate governance [20]. Khan and Kamal [36] reported that in Pakistan via CCG-2017 the CEO duality in the listed firms of PSX has been withdrawn to strengthen the BOD functions. In the extant literature, two views are associated with CEO duality and EM. One perspective is the agency theory perspective, which states that the CEO and chairman roles should be separated for effective monitoring. According to Guluma [20], the dual role of CEO minimizes board independence, enhances the CEO’s entrenchment and results in poor financial performance of a firm, which stimulates the management to engage in EM. Moreover, separation of roles between CEO and chairman is essential because it enables the management to influence accounting policies (EM), which benefit them at the expense of minority shareholders [73]. The other perspective or view is the stewardship theory perspective. This point of view on CEO duality contends that it reduces agency conflict, promotes clear direction of the firm for development, and unites and strengthens leadership within the firm [20]. Thus, CEOs in dual positions are good stewards of the owners, work for the best interests of the firms, and are not involved in EM practices. In the extant literature, mixed results are available. The dual position of CEO results in a greater magnitude of EM and may cause mis-reporting earnings to maintain their status quo [5, 7, 30]. However, in the CCG-2017 in Pakistan, the duality of the CEO has been withdrawn. Now, in Pakistan, a person cannot hold the positions of CEO and chairman at the same time. Therefore, in order to check the influence of new regulations regarding the CEO duality, this research study proposes the following hypothesis,

#### H4:

CEO duality is more effective in limiting earnings management (accrual and real EM) in Pakistani non-financial listed firms in the post-CCG-2017 period than in the pre-CCG-2017 period.

## Methodology

### Sample selection and data

The population of this study is the non-financial listed firms of PSX. The initial sample for this study was 543 listed firms. The financial firms (132) have been excluded due to the difference of accruals procedure and accounting policies as mentioned by [8, 33, 63] in the concerned literature. In addition to this, companies with missing annual reports and defaulter companies and those companies which were delisted were also excluded (86); furthermore, 10 companies were those whose board of directors information were not given. The remaining (323) companies’ data were used in this study for the observable period of 2015–2019 consisting of 1114 to 1607 firm-year observations due to the unbalance panel. The sample firms are representative of the PSX because it is about 77% of the listed non-financial firms.

The detail about sample selection procedures and sample distribution is given in Tables 1 and 2. The final sample consists the data of 27 sectors of PSX excluding the financial sectors. The study period is from 2015 to 2019. The logic behind this period is to take two years the pre- and post-period of the CCG-2017 in order to check CCG-2017 impact on EM of the pre- and post-period. The pre-period is 2015 and 2016, while the post-period is 2018 and 2019. The data of this research study have manually collected from the annual reports of the companies’, concerned companies’ websites and the State Bank of Pakistan sources/publication. Furthermore, the data used in this research study are part of the data published in the study of [38].

**Table 1:** Procedures of sample selection

Total firms listed in the PSX in June 2021	551
Less: Financial firms	(132)
	419
Less: Annual reports missing, defaulter and delisted firms for the said period	(86)
	333
Less: Firms with missing Board of Directors and Audit Committee information	(10)
Firms selected from the for the study	323

**Table 2:** Sample distribution by year and industry

S. no.	PSX sector name	Code	Year					Total by Industry	%
			2015	2016	2017	2018	2019		
1	Automobile Assembler	801	12	12	12	12	12	60	3.73
2	Automobile Parts andAccessories	802	7	7	7	7	7	35	2.18
3	Cable and Electrical Goods	803	5	5	5	5	5	25	1.56
4	Cement	804	20	20	20	20	20	100	6.22
5	Chemical	805	27	27	27	27	27	135	8.40
6	Engineering	808	11	11	11	11	11	55	3.42
7	Fertilizer	809	5	5	5	5	5	25	1.56
8	Food and Personal Products	810	17	17	17	17	17	85	5.29
9	Glass and Ceramics	811	8	8	8	8	8	40	2.49
10	Leather and Tanneries	816	1	1	1	1	1	5	0.31
11	Miscellaneous	818	10	10	10	10	10	50	3.11
12	Oil and Gas Exploration Co	820	4	4	4	4	4	20	1.24
13	Oil and Gas Marketing Co	821	8	8	8	8	8	40	2.49
14	Paper and Board	822	8	8	8	8	8	40	2.49
15	Pharmaceuticals	823	10	10	10	10	10	50	3.11
16	Power Generation and Distribution	824	14	14	14	14	14	70	4.36
17	Refinery	825	5	5	5	5	5	25	1.56
18	Sugar and Allied Industries	826	29	29	29	29	29	145	9.02
19	Synthetic and Rayon	827	8	8	8	8	8	40	2.49
20	Technology and Communication	828	13	13	13	13	13	65	4.04
21	Textile Composite	829	54	54	54	54	54	270	16.80
22	Textile Spinning	830	32	31	31	32	31	157	9.77
23	Textile Weaving	831	5	5	5	5	5	25	1.56
24	Tobacco	832	2	2	2	2	2	10	0.62
25	Transport	833	4	4	4	4	5	21	1.31
26	Vanaspati and Allied Industries	834	2	2	2	2	1	9	0.56
27	Woolen	835	1	1	1	1	1	5	0.31
			322	321	321	322	321	**1607**	
	**Total**		**20.04**	**19.98**	**19.98**	**20.04**	**19.98**		**100**

### Variables measurement

#### Earnings management proxies

In the earnings management and related literature, the most used proxy for EM is discretionary accruals estimated under Jones Model [32]. The Jones Model developed by [32] is considered the standard model of discretionary accruals because every other model of discretionary accruals has been established by modifying the Jones Model; studies which extended Jones Model are [13, 14, 42, 48]. In this research study, McNichols, [48] model will be used because her model is the combination of accruals and cash flow components; she combines the Jones Model [32] and Dechow and Dichev Model [13]. The McNichols [48] model of EM is estimated as in Eq. 1.

1 $$\begin{aligned} \frac{{ACC_{IT} }}{{LAGTA_{IT - 1} }} & = \beta_{o} + \beta_{1} \left( {CFO_{IT - 1} {|}LAGT_{IT - 1} } \right) + \beta_{2} \left( {CFO_{IT} {|}LAGTA_{IT - 1} } \right) \\ & \quad + \beta_{3} \left( {CFO_{IT + 1} {|}LAGTA_{IT - 1} } \right) \beta_{4} \left( {{\Delta }SALES_{IT} {|}LAGTA_{IT - 1} } \right) + \beta_{5} \left( {PPE_{IT} {|}LAGTA_{IT - 1} } \right) + \epsilon_{IT} \\ \end{aligned}$$

where ACC_IT_ is the accruals as calculated by subtracting cash flow from operation from net income after tax as reported in the annual report; CFO_IT_, CFO_IT-1_, CFO_IT+1_ means cash flow from operation for t year, cash flow from operation for year t + 1 and t-1. Moreover, ${\Delta SALES}_{IT}$ is change in sales in t year, PPE_IT_ is the property plant and equipment for year t and ${LAGTA}_{IT-1}$ is the lagged value of total assets in year t.

Moreover, in this research study Real Activity Earnings Manipulation (hereafter, REM) proxies have also used in order to check its relationship with the BOD-related variables. This study followed [58] for REM proxies, which are calculated in the following equations:

2 $$\frac{{CFO_{IT} }}{{LAGTA_{IT} }} = \; \beta_{o} + \beta_{1} \left( {1{|}LAGTA_{IT} } \right) + \beta_{2} \left( {SALES_{IT} {|}LAGTA_{IT} } \right) + \beta_{3} \left( {{\Delta }SALES_{IT} {|}LAGTA_{IT} } \right) + \epsilon_{IT}$$

3 $$\frac{{PROD_{IT} }}{{LAGTA_{IT} }} = \; \beta_{o} + \beta_{1} \left( {1{|}LAGTA_{IT} } \right) + \beta_{2} \left( {SALES_{IT} {|}LAGTA_{IT} } \right) + \beta_{3} \left( {{\Delta }SALES_{IT} {|}LAGTA_{IT} } \right) + \beta_{2} \left( {{\Delta }SALES_{IT - 1} {|}LAGTA_{IT} } \right) + \epsilon_{IT}$$

4 $$\frac{{DISEXP_{IT} }}{{LAGTA_{IT} }} = \; \beta_{o} + \beta_{1} \left( {1{|}LAGTA_{IT} } \right) + \beta_{2} \left( {SALES_{IT} {|}LAGTA_{IT} } \right) + \epsilon_{IT}$$

### Hypothesis testing

In order to test the study’s hypotheses, the following equation will be used,

5 $$DRM_{it} = \; \theta_{^\circ } + \theta_{1} BIND_{it} + \theta_{2 } BEXP_{it} + \theta_{3} BDIV_{it} + \theta_{4} CEOD_{it} + \mathop \sum \limits_{M} pm\,Control + \mu_{it}$$

6 $$REM_{it} = \; \theta_{^\circ } + \theta_{1} BIND_{it} + \theta_{2 } BEXP_{it} + \theta_{3} BDIV_{it} + \theta_{4} CEOD_{it} + \mathop \sum \limits_{M} pm\,Control + \mu_{it}$$

In Eqs. 5 and 6, the dependent variables DRM is discretionary accrual calculated under [48] model, while REM is the four proxies calculated under [58] model. The predictors are: BIND is for board independence, BEXP is for board expertise and BDIV is for the female directorship, CEOD is for the dual position of CEOs, control is for a battery of control variables; the detail is fully given in appendix Table 15.

## Empirical results

The objective of the study is to assess whether the newly mentioned board of directors’ directions in CCG-2017 regarding BOD characteristics are influential in changes in management EM practices as proxied by accruals and REM. The data used for this study are the panel (unbalanced), so various diagnostic tests are made prior to panel data estimation. Table 3 gives us the results of multicollinearity, heteroscedasticity, and autocorrelation. The results of the tests show that there is no issue of multicollinearity and autocorrelation, but there is an issue of heteroscedasticity, which is handled by robust standard error in regression estimation.

**Table 3:** The diagnostic tests before regression

Variance inflation factor										
Variables	ROA	BEXP	ACEXP	LOSS(D)	BIND	FSIZE	ACIND	SALES	BIG4	INV
VIF	2.65	2.05	1.92	1.87	1.73	1.65	1.64	1.63	1.56	1.53
1/VIF	0.38	0.49	0.52	0.54	0.58	0.61	0.61	0.61	0.64	0.65
Variables	LEVE	TANG	FD	BSIZE	BF	BMEET	FAGE	CEOD	CFO	
VIF	1.49	1.34	1.26	1.23	1.18	1.18	1.09	1.03	1.01	
1/VIF	0.67	0.75	0.79	0.81	0.85	0.85	0.92	0.97	0.99	

Breusch–Pagan/Cook–Weisberg test for heteroscedasticity	
Ho: Constant variance Variables: fitted values of MCDA χ2 (1) = 5.26 χ2 > chi2 = 0.0219	

Wooldridge test for autocorrelation	
H0: no first-order autocorrelation F ( 1, 197) = 0.279 F-Statistics = 0.5980	

### Descriptive statistics

#### Year wise descriptive statistics

In Table 4, the year-wise descriptive statistics have been given. The results show that the number of independent directors on the board has increased from 16 percent in 2015 to 21 percent in 2019. This figure indicates that board independence has increased significantly since the implementation of CCG-2017. Financial expert directors, like board independence, increased from 31 to 35 percent between 2015 and 2019. In addition to this, the presence or proportion of female directors in the board room has slightly increased from 10 to 12 percent. Moreover, board size and meetings are almost the same in all years, while the dual position of CEO has decreased from 4 to 1 percent in all the years.

**Table 4:** Year-wise descriptive statistics of board of directors, and earnings management

Variables	Year 2015			Year 2016			Year 2017			Year 2018			Year 2019		
	N	Mean	SD	N	Mean	SD	N	Mean	SD	N	Mean	SD	N	Mean	SD
*Board of directors’ variables*															
**BIND**	**308**	**.167**	**.076**	**307**	**.165**	**.074**	**313**	**.17**	**.076**	**310**	**.19**	**.083**	**306**	**.213**	**.085**
**BEXP**	**223**	**.313**	**.143**	**222**	**.328**	**.147**	**224**	**.338**	**.151**	**223**	**.341**	**.155**	**222**	**.354**	**.155**
**BDIV**	**310**	**.101**	**.155**	**309**	**.098**	**.146**	**311**	**.1**	**.139**	**310**	**.109**	**.14**	**306**	**.127**	**.136**
FD	305	.298	.23	302	.293	.231	306	.288	.231	304	.28	.229	300	.278	.229
BMEET	308	5.26	2.12	308	5.38	2.01	310	5.42	1.98	307	5.37	1.84	287	5.27	1.84
BSIZE	311	7.78	1.05	309	7.79	1.05	313	7.79	1.07	311	7.79	1.05	307	7.78	1.07
**CEOD**	**310**	**.042**	**.201**	**309**	**.074**	**.55**	**312**	**.029**	**.168**	**311**	**.013**	**.113**	**306**	**.013**	**.114**
*Dependent variable (Discretionary accruals)*															
**MCDA**	**292**	**−.039**	**.103**	**298**	**.008**	**.088**	**299**	**.023**	**.114**	**300**	**.021**	**.116**	**287**	**.001**	**.13**
**REALEM**	**284**	**.182**	**.313**	**295**	**.147**	**.299**	**300**	**.078**	**.267**	**305**	**.025**	**.271**	**308**	**.069**	**.256**
DIS ERM	299	.153	.403	304	.152	.474	307	.162	.625	309	.118	.413	314	.171	.631
RCFO	297	.039	.139	301	.016	.132	304	−.027	.134	307	−.043	.104	309	−.02	.11
PRO RM	286	.039	.179	295	.028	.176	303	.002	.154	306	−.024	.147	308	−.016	.149
*Control variables*															
ACIND	305	.271	.148	306	.285	.134	311	.3	.12	311	.319	.118	307	.33	.10
ACEXP	225	.315	.248	222	.324	.244	223	.342	.253	223	.357	.246	223	.37	.25
BIG4	319	.486	.501	319	.492	.501	319	.483	.5	320	.475	.5	318	.47	.5
ROA	304	4.62	9.09	309	5.262	9.05	309	5.43	8.63	314	4.99	8.33	310	4.44	8.45
LEVE	304	.542	.219	309	.531	.222	309	.541	.22	314	.556	.22	310	.55	.217
FAGE	323	35.98	15.35	322	36.91	15.36	322	37.59	15.37	323	38.47	15.27	322	39.28	15.22
TANG	302	.46	.221	309	.461	.222	309	.449	.22	314	.446	.225	310	.44	.229
FSIZE	304	8.45	1.34	309	8.517	1.35	308	8.61	1.37	314	8.66	1.41	310	8.71	1.42
LOSS(D)	319	.307	.462	318	.283	.451	321	.252	.435	320	.263	.441	313	.27	.44
CFO	297	.065	.26	301	.063	.129	306	.033	.176	305	−1.20	21.50	308	.03	.14
SALES	304	1.02	.761	308	.944	.663	309	.784	1.94	314	.886	.68	310	.89	.70
INV	302	.146	.122	309	.141	.122	309	.137	.288	314	.151	.131	310	.14	.13

The dependent variable of the study EM (MCDA), which is discretionary accruals calculated under the [48] model, has decreased from 2015 to 2019. The RAM EM proxy calculated under the [58] method has also decreased from 2015 (0.18) to 2019 (0.069). This finding of our research study shows that after the implementation of CCG-2017 EM has decreased in PSX non-financial listed firms.

In Table 5, full-sample summary statistics are given. The results of Table 5 show that among BOD variables, board independence has an average of 18 percent in Pakistani listed firms, which is according to the new compulsory directions. According to it, if there is a 7-member board, there must be 2 independent directors, which is 14 percent of the board. The average board size is a minimum of 7 directors in the board in Table 5, which justifies the 18 percent independence. The firms with financially expert directors account for 33 percent of the non-financial listed firms in Pakistan, while the minimum number is 14 percent and 57 percent is the maximum. Female representation in the board is 10 per cent on average in Pakistani listed non-financial firms. When it comes to CEO duality, it has steadily decreased in recent years, accounting for 3% on average in 77% of Pakistan’s non-financial listed firms. The aggregate proxy of REM manipulation, the predictor variable in this sample, has 0.09 mean value, while −2.39 and.967 are the minimum and maximum values. The discretionary accruals proxy MCDA, on the other hand, has a mean value of 0.05, with minimum and maximum values of −0.759 and.992, respectively.

**Table 5:** Full-sample descriptive statistics

Variables	N	Mean	Median	SD	Min	Max
*Predictor variables*						
MCDA	1476	0.05	−.001	.113	−.759	.992
REALEM	1492	.099	.063	.286	−2.39	.967
DIS ERM	1533	.151	.086	.52	−3.00	9.102
RCFO	1518	−.008	−.006	.128	−.78	.867
PROD RM	1498	.005	.004	.163	−.256	.285
*Board of directors variables*						
**BIND**	**1544**	**.181**	**.143**	**.081**	**.091**	**.333**
**BEXP**	**1114**	**.335**	**.286**	**.151**	**.143**	**.571**
**BDIV**	**1546**	**.107**	**0**	**.144**	**0**	**1**
FD	1517	.287	.286	.23	0	.625
BMEET	1520	5.346	5	1.96	2	24
BSIZE	1551	7.792	7	1.06	7	10
**CEOD**	**1548**	**.034**	**0**	**.282**	**0**	**1**
*Control variables*						
ACIND	1540	.303	.333	.129	0	.5
ACEXP	1116	.343	.333	.25	0	.75
BIG4	1595	.483	0	.5	0	1
ROA	1546	4.95	4.08	8.71	−8.80	19.89
LEVE	1546	.545	.543	.22	.213	.92
FAGE	1607	37.65	35	15.34	15	63
TANG	1544	.452	.456	.224	.101	.797
FSIZE	1545	8.59	8.55	1.38	6.373	10.7
LOSS(D)	1591	.275	0	.447	0	1
CFO	1517	−.204	.028	9.64	−375.50	1.55
SALES	1545	.905	.843	1.07	−31.29	5.74
INV	1544	.144	.136	.171	−4.37	.651

### Mean differences test of the pre- and post-period of CCG-2017

In the Table 6 pre and post year descriptive statistics as well as their T-Test of mean differences is given in detail. In the BODs attributes board independence, board diversity, CEO Duality and board expertise is significantly different in the pre and post periods. Board independence as expected significantly increase in the post period as compare to pre period up-to 3 per cent. Similarly, gender diversity has significantly increased in the post period and the difference between pre and post period is almost 2 percent which is expected according to our predictions. In addition to this, expertise in board has also significantly increased in the post period as compare to the pre-period by 2 percent in Pakistani listed firms. On the other hand, the predicators of EM has also significantly different in the post period from the pre period of CCG-2017.

**Table 6:** T-Test for the differences in mean of pre- and post-period of CCG-2017

Variables	Panel A			Panel B			Panel C mean differences		
	Pre-Period (2015 &2016)			Post-Period (2018 & 2019)			Mean difference panel (A-B)	T-Value	P-Value
	N	Mean	SD	N	Mean	SD			
**BIND**	**615**	**.166**	**1.52**	**616**	**.201**	**1.53**	**−.035**	**−7.7**	0***
**BEXP**	**445**	**.321**	**0.00**	**445**	**.347**	**0.00**	**−.027**	**−2.65**	.008***
**BDIV**	**619**	**.100**	**.150**	**616**	**.119**	**0.13**	**−.018**	**−2.25**	.024***
FD	607	.296	1.90	604	.279	1.84	.017	1.3	.193
BMEET	616	5.32	0.20	594	5.32	0.10	.004	.05	.967
BSIZE	620	7.79	1.05	618	7.78	1.06	0.01	0	.991
**CEOD**	**619**	**058**	**.413**	**617**	**0.01**	**.113**	**0.04**	**2.60**	.009***
*Dependent variables*									
**MCDA**	**590**	**−.024**	**.167**	**587**	**.012**	**.176**	**−.035**	**−5.4**	0***
**REALEM**	**579**	**.165**	**.132**	**613**	**.047**	**.119**	**.117**	**7.1**	0***
DIS ERM	603	.153	.241	623	.144	.248	.008	.3	.776
RCFO	598	.027	.130	616	−.032	.104	.059	8.4	0
PRO RM	581	.034	.177	614	−.02	.147	.053	5.65	0
*Control variables*									
ACIND	611	.278	0.16	618	.329	0.174	−.051	−6.95	0
ACEXP	447	.32	0.27	446	.367	0.278	−.048	−2.9	.004
BIG4	638	.500	.49	638	.476	.019	.013	.45	.654
ROA	613	4.94	9.07	624	4.70	8.39	.241	.5	.628
LEVE	613	.536	.220	624	.556	.218	−.02	−1.6	.106
FAGE	645	36.44	15.35	645	38.87	15.23	−2.43	−2.85	.004
TANG	611	.460	.221	624	.445	.227	.015	1.2	.235
FSIZE	613	8.48	1.35	624	8.68	1.41	−.203	−2.6	.01
LOSS(D)	637	.442	1.51	633	.267	.456	.025	1.1	.265
CFO	598	.064	.204	613	−.584	.612	.647	1.05	.297
SALES	625	16,941	.713	624	23,481.5	.693	−6539.9	2.4	.018
INV	611	.143	.121	624	.149	.130	−.005	−.8	.433

### Correlation matrix

Table 7 displays correlations of the study variables. As expected, there are negative significant correlations between board independence and expertise with EM (MCDA), while the relationship of female directors and EM is positive and significant. Furthermore, the correlation between EM proxies and board independence and expertise with audit committee independence and expertise is high and positive, which is theoretically correct. Furthermore, all the values are under 0.07, which shows that there is no issue of multicollinearity.

**Table 7:** Pairwise correlation matrix

Variables	MCDA	REM	DISERM	RCFO	P_RM	BIND	BEXP	BDIV	FD	BMEET	BSIZE	ACIND	ACEXP	BIG4	ROA	LEVE	FAGE	TANG	FSIZE	LOSS(D)	CEOD	CFO	SALES	INV
MCDA	1																							
REM	−0.06*	1																						
DIS_ERM	−0.00	0.25*	1																					
RCFO	−0.25*	0.73*	−0.00	1																				
P_RM	0.04	0.82*	0.00	0.58*	1																			
BIND	0.01	−0.09*	0.01	−0.06*	−0.12*	1																		
BEXP	−0.03	−0.11*	−0.00	−0.06*	−0.11*	0.19*	1																	
BDIV	−0.00	0.06*	0.02	0.06*	0.03	−.04*	−.17*	1																
FD	0.00	0.11*	−0.00	0.07*	0.14*	−.19*	−0.00	0.24*	1															
BMEET	0.02	−0.02	−0.03	−0.03	0.01	0.11*	−.09*	0.03	−.07*	1														
BSIZE	−0.00	−0.13*	−0.03	−0.07*	−0.10*	0.04*	−0.01	−.09*	−.20*	0.09*	1													
ACIND	0.04	−0.11*	−0.00	−0.11*	−0.08*	0.56*	0.07*	−.05*	−.12*	0.09*	0.06*	1												
ACEXP	−0.00	−0.12*	0.03	−0.08*	−0.10*	0.16*	0.67*	−.18*	−.00	−0.08*	0.13*	0.06*	1											
BIG4	−0.03	−0.25*	−0.05*	−0.11*	−0.26*	0.14*	0.28*	−.16*	−.25*	0.04	0.26*	0.09*	0.24*	1										
ROA	−0.06*	−0.47*	−0.07*	−0.28*	−0.57*	0.07*	0.20*	−.12*	−.14*	0.02	0.20*	0.08*	0.15*	0.41*	1									
LEVE	0.01	0.19*	0.00	0.0*	0.29*	0.00	−.15*	0.06*	−0.03	0.03	−0.09*	−0.01	−0.13*	−.21*	−.50*	1								
FAGE	0.00	−0.05*	−0.01	−0.02	−0.07*	0.06*	0.10*	−.08*	−0.02	0.02	0.14*	0.08*	0.09*	0.07*	0.07*	−.13*	1							
TANG	−0.05	0.10*	−0.07*	0.08*	0.15*	−0.03	−.17*	0.15*	0.16*	−0.04*	−0.15*	−0.01	−0.16*	−.24*	−.41*	0.26*	−0.10*	1						
FSIZE	−0.00	−0.34*	−0.16*	−0.19*	−0.17*	0.10*	0.24*	−.23*	−.20*	0.15*	0.27*	0.12*	0.21*	0.44*	0.35*	−.043	0.08*	−0.16*	1					
LOSS(D)	0.033	0.365*	0.06*	0.22*	0.38*	−0.01	−.15*	0.05*	−0.01	−0.04	−0.15*	−0.04	−0.12*	−.29*	−.64*	0.39*	−0.04*	0.32*	−0.33*	1				
CEOD	−0.00	0.02	−0.00	0.02	0.03	−.06*	−0.00	0.07*	0.07*	−0.00	−0.06*	−0.10*	−0.01	−.07*	−.05*	0.00	−0.00	0.06*	−0.06*	0.03	1			
CFO	−.239*	−0.02	0.11*	0.07*	−0.02	−0.00	−0.01	−0.00	0.00	−0.01	0.01	−0.01	0.00	0.03	0.04	−0.00	−0.02	0.03	0.04	−0.01	−0.00	1		
SALES	0.005	0.02	−0.03	0.02	−0.01	−0.04	0.08*	−0.02	−0.04	−0.00	0.09*	−0.05*	0.08*	0.14*	0.21*	0.01	0.03	−0.13*	0.11*	−0.17*	0.00	−.00	1	
INV	0.100*	0.06*	−0.04*	0.02	0.06*	−.07*	0.02	−0.03	0.01	−0.05*	0.07*	−0.04*	0.07*	0.06*	0.09*	0.03	0.08*	−0.18*	0.00	−0.12*	−0.02	−.02	0.73*	1

*Shows significance at the 0.05 level

### Regression results

Table 8 reports the main results of this study, which examines the board of directors’ characteristics on EM. Before the estimation, we carried out the Hausman test to choose between fixed affect and random affect.

**Table 8:** Regression results of board of directors attributes and earnings management (Discretionary Accruals)

Dependent Variable	(1)		(2)		(3)	
	Pre-Period (2015 and 2016)		Post-Period (2018 and 2019)		Full-Period	
	MCDA		MCDA		MCDA	
Independent Variables	Coefficient	t-value	Coefficient	t-value	Coefficient	t-value
**BIND**	**−0.146**	**(−1.23)**	−0.450^**^	**(−1.65)**	−0.224^***^	**(−2.33)**
**BEXP**	**0.051**	**(0.54)**	−0.454^***^	**(−2.02)**	−0.137^**^	**(−1.78)**
**CEOD**	**−0.002**	**(−0.24)**	**0.000**	**(.)**	**0.007**	**(0.54)**
**BDIV**	**0.039**	**(0.40)**	**0.277**	**(0.99)**	0.169^***^	**(2.05)**
FD	0.111	(0.86)	−0.170	(−0.47)	−0.116	(−1.20)
BMEET	0.008^**^	(1.81)	0.002	(0.23)	0.000	(0.06)
BSIZE	0.010	(0.75)	−0.033	(−0.79)	0.009	(0.75)
ACIND	−0.042	(−0.67)	0.246	(1.00)	0.133^***^	(2.20)
ACEXP	−0.071	(−1.09)	0.112	(0.94)	−0.003	(−0.06)
BIG4	−0.012	(−0.27)	0.005	(0.05)	0.023	(0.68)
ROA	0.002	(1.48)	−0.002	(−0.64)	−0.001	(−0.85)
LEVE	−0.165^***^	(−2.30)	0.004	(0.02)	0.002	(0.04)
FAGE	0.016^***^	(2.45)	−0.016	(−0.85)	0.011^***^	(2.88)
TANG	0.012	(0.19)	−0.058	(−0.33)	0.015	(0.30)
FSIZE	0.062^**^	(1.80)	0.089	(1.02)	0.047^***^	(2.34)
LOSS(D)	0.024	(1.54)	0.048	(1.16)	0.043^***^	(2.79)
CFO	−0.911^***^	(−21.88)	−0.001	(−1.63)	−0.002^***^	(−4.61)
SALES	0.046^***^	(2.58)	0.128^***^	(2.21)	0.031^**^	(1.73)
INV	−0.251^***^	(−2.22)	0.651^***^	(2.84)	0.553^***^	(6.84)
Constant	−1.162^***^	(−3.14)	0.055	(0.06)	−0.977^***^	(−4.89)
Observations	394		381		977	
*Groups(firms)*	207		205		217	
*R*^2^	0.778		0.170		0.188	
F-Statistics	31.343***		1.801***		9.042***	

*t* statistics in parentheses, ^**^ *p* < 0.10, ^***^ *p* < 0.05

Table 9 shows with a *P*-value of (0.000) that the fixed effect is the best model. The regression is carried out with robust standard errors. The findings of this study show that there is a significant relationship of board independence and financially expert directors with EM in the post-CCG-2017 period and full regression. The coefficient and the corresponding t-value of board independence and financial expert directors are −0.450**(−1.65) and −0.454***(−2.02) for the post-period and −0.22***(−2.33) and −0.13** (−1.78) for full-period regression. Moreover, in the pre-period, there is no significant relationship between board independence and expert directors with EM (Table 10).

**Table 9:** Hausman (1978) specification test

Test summary	Coef
Chi-square test value	108.665
*P*-value	0.000

**Table 10:** Regression results of board of directors attributes and earnings management with the pre- and post-CCG 2017 dummies

Dependent Variable	(4)		(5)		(6)	
	Pre-Period (2015&2016)		Post-Period (2018&2019)		Full-Period	
	MCDA		MCDA		MCDA	
Independent variables	Coefficient	t-value	Coefficient	t-value	Coefficient	t-value
BIND_PRE_17	− 0.143	(− 1.21)				
BEXP_PRE_17	0.053	(0.56)				
BDIV_PRE_17	0.045	(0.38)				
FD_PRE_17	0.109	(0.84)				
CEOD_PRE_17	0.000	(0.01)				
**BIND_POST_17**			−0.448^**^	**(−1.64)**		
**BEXP_POST_17**			−0.454^***^	**(−2.01)**		
BDIV_POST_17			0.282	(0.98)		
FD_POST_17			−0.171	(−0.47)		
CEOD_POST_17			0.000	(.)		
**BIND**					−0.228^***^	**(−2.37)**
**BEXP**					−0.136^**^	**(−1.77)**
**BDIV (%)**					0.174^***^	**(2.11)**
FD					−0.116	(−1.20)
CEOD					0.058	(1.26)
BMEET	0.008^**^	(1.79)	0.002	(0.23)	0.000	(0.04)
BSIZE	0.010	(0.75)	−0.032	(−0.78)	0.010	(0.86)
ACIND	−0.042	(−0.67)	0.244	(1.00)	0.138^***^	(2.28)
ACEXP	−0.071	(−1.09)	0.112	(0.95)	−0.002	(−0.06)
BIG4	−0.011	(−0.26)	0.006	(0.05)	0.023	(0.68)
ROA	0.002	(1.47)	−0.002	(−0.62)	−0.001	(−0.78)
LEVE	−0.164^***^	(−2.28)	0.005	(0.03)	0.006	(0.10)
FAGE	0.016^***^	(2.43)	−0.016	(−0.85)	0.011^***^	(2.93)
Tangibility	0.012	(0.19)	−0.056	(−0.32)	0.020	(0.38)
FSIZE	0.062^**^	(1.79)	0.088	(1.01)	0.047^***^	(2.31)
LOSS(D)	0.024	(1.54)	0.048	(1.17)	0.044^***^	(2.87)
CFO	−0.911^***^	(−21.8)	−0.001	(−1.57)	−0.002^***^	(−4.61)
SALES	0.046^***^	(2.58)	0.127^***^	(2.20)	0.030^**^	(1.70)
INV	−0.251^***^	(−2.22)	0.652^***^	(2.84)	0.555^***^	(6.87)
Constant	−1.162^***^	(−3.12)	0.060	(0.06)	−0.996^***^	(−4.97)
Observations	393		381		976	
Groups (Firms)	205		205		217	
*R*^2^	0.77		0.17		0.18	
F-Statistics	31.08***		1.80**		9.12***	

*t* statistics in parentheses, ^**^ *p* < 0.10, ^***^ *p* < 0.05

Therefore, the H1 and H3 of the study are accepted, which state that independent and financially expert directors are more effective in limiting EM practices in the post-CCG-2017 period as compared to the pre-CCG-2017 period.

The findings of this study demonstrate that outside/independent directors reduce earnings manipulation, which is consistent with the studies of [41, 63, 76, 78]. Cheng, Chen, and Wang (2015) added that independent directors reduce EM in situations where the acquisition of company-related information is easy and cost-effective. In addition to this, Young et al., [78] posited that those boards that have more independent and professional directors have a negative impact on earnings manipulation, which result in better board governance and monitoring. The literature review study [46] indicated that board independence in the board improves the board’s (management) ability to monitor and control management’s EM practices.

Furthermore, Khan and Kamal [36] found that companies with professional and previous working experience directors on their boards will reduce earnings manipulation. The director’s professional expertise, especially accounting expertise, is one of the crucial feature in their monitoring role [55]. This quality of directors constrains management from EM. Moreover, the study by [67] posited that for the monitoring process and to make the financial statements more transparent, directors must have the expertise (accounting cum past working experience) that will enable them to curb EM. These study’s findings are similar to the findings of the above-mentioned studies. Therefore, this study showed evidence that professional and expert directors reduced EM in Pakistani listed firms.

In addition to board independence and board expertise, the coefficients and t-values of female directors (**BDIV**) and CEO duality (**CEOD**) indicate that female directors and CEO duality are not related to EM in the post-period of CCG-2017. Moreover, the female directors and CEO duality are also not significant in the pre-period of CCG-2017. However, the female directors are positively related to EM in the full-period regression. These findings are in line with [68]. Although the outcomes of this research contradict the study by [72] in the Pakistani context, which posited that diversity in corporate boards in Pakistan reduces EM. Furthermore, they added that female directors are very viable in board rooms and their presence in the board strengthens the effectiveness of the BOD, which curtails EM. These results depict that the study’s hypotheses H2 and H4 cannot be accepted. In control variables in the full-period regression, the firm age (FAGE), firm size (FSIZE) and loss dummy (LOSS D) are positively related to EM.

In addition to this, BOD characteristics such as independence, female directors and financial expert directors, which were also the point of concern in the CCG, are also checked and related to REM reported in Table 11 and 12. All the outcome variables show insignificant relationships with REM proxies such as aggregate REM by adding abnormal cash flow from operations, abnormal discretionary expenses, and production costs, as well as individually with each of the aforementioned proxies of REM. Moreover, as evident in Tables 11 and 12, the female directors are positively related to aggregate REM and abnormal cash flow from operations.

**Table 11:** Regression of board of director attributes and real activity manipulation

Dependent Variable	(7) Pre-Period (2015&2016)		(8) Post-Period (2018&2019)		(9) Full-Period		(10) Pre-Period (2015&2016)		(11) Post-Period (2018&2019)		(12) Full-Period	
	RM		RM		RM		RMCFO		RMCFO		RMCFO	
Independent Variables	Coefficient	t-value	Coefficient	t-value	Coefficient	t-value	Coefficient	t-value	Coefficient	t-value	Coefficient	t-value
**BIND**	−0.783^**^	**(−1.93)**	**0.106**	**(0.26)**	**0.086**	**(0.47)**	**−0.359**	**(−1.65)**	**0.064**	**(0.45)**	**0.002**	**(0.02)**
**BEXP**	**−0.396**	**(−1.20)**	**0.116**	**(0.34)**	**−0.127**	**(−0.85)**	**−0.022**	**(−0.13)**	**0.200**	**(1.62)**	**−0.120**	**(−1.51)**
**CEOD**	**−0.094**	**(−0.54)**	**0.000**	**(.)**	−0.180^***^	**(−2.02)**	**−0.039**	**(−0.41)**	**0.000**	**(.)**	**0.000**	**(0.01)**
**BDIV**	**0.490**	**(1.48)**	**−0.188**	**(−0.46)**	0.290^**^	**(1.84)**	**0.178**	**(1.02)**	**−0.039**	**(−0.27)**	0.174^***^	**(2.08)**
FD	0.725^**^	(1.66)	−0.116	(−0.25)	0.323^**^	(1.78)	0.215	(0.91)	−0.000	(−0.00)	0.202^***^	(2.09)
BMEET	−0.019	(−1.28)	0.001	(0.03)	0.002	(0.24)	−0.008	(−1.05)	−0.002	(−0.31)	−0.002	(−0.64)
BSIZE	0.040	(0.81)	0.012	(0.23)	−0.003	(−0.16)	0.031	(1.23)	0.014	(0.72)	0.012	(1.07)
ACIND	−0.050	(−0.23)	0.091	(0.25)	−0.095	(−0.81)	0.030	(0.26)	0.127	(0.99)	−0.136^***^	(−2.21)
ACEXP	−0.293	(−1.25)	0.018	(0.10)	0.086	(1.02)	−0.103	(−0.87)	0.009	(0.15)	0.021	(0.48)
BIG4	−0.146	(−0.99)	−0.057	(−0.33)	0.012	(0.19)	−0.032	(−0.40)	−0.061	(−0.98)	−0.026	(−0.75)
ROA	0.000	(0.08)	−0.013^***^	(−2.78)	−0.016^***^	(−7.46)	−0.002	(−0.81)	−0.007^***^	(−3.87)	−0.006^***^	(−5.21)
LEVE	0.149	(0.59)	−0.133	(−0.50)	−0.252^***^	(−2.36)	0.135	(1.03)	−0.179^**^	(−1.87)	−0.124^***^	(−2.20)
FAGE	−0.042^**^	(−1.82)	0.096^***^	(3.36)	−0.028^***^	(−3.78)	−0.020	(−1.65)	0.035^***^	(3.46)	−0.015^***^	(−3.94)
Tangibility	0.148	(0.67)	0.010	(0.04)	0.075	(0.75)	−0.042	(−0.36)	0.099	(1.01)	−0.043	(−0.82)
FSIZE	−0.465^***^	(−3.94)	−0.447^***^	(−3.44)	−0.213^***^	(−5.45)	−0.225^***^	(−3.55)	−0.262^***^	(−5.67)	−0.052^***^	(−2.53)
LOSS(D)	0.001	(0.03)	−0.013	(−0.21)	−0.000	(−0.01)	0.000	(0.02)	−0.021	(−0.96)	−0.007	(−0.43)
CFO	−0.174	(−1.23)	0.000	(0.19)	−0.001	(−1.35)	−0.169^***^	(−2.23)	0.002^***^	(5.27)	0.000	(0.70)
SALES	−0.641^***^	(−10.55)	−0.366^***^	(−4.09)	−0.282^***^	(−8.16)	−0.224^***^	(−6.89)	−0.176^***^	(−5.54)	−0.086^***^	(−4.68)
INV	1.680^***^	(4.35)	2.052^***^	(5.84)	1.367^***^	(8.83)	0.377^**^	(1.82)	0.527^***^	(4.22)	0.248^***^	(3.01)
Constant	6.015^***^	(4.67)	0.188	(0.13)	3.205^***^	(8.19)	2.706^***^	(4.01)	0.851^**^	(1.67)	1.133^***^	(5.52)
N	384		397		980		395		398		996	
Groups (Firms)	203		209		218		205		209		218	
*R*^2^	0.555		0.352		0.307		0.383		0.417		0.177	
F-Statistics	10.686***		5.140***		17.309***		5.615***		6.796***		8.608***	

*t* statistics in parentheses, ^**^ *p* < 0.10, ^***^ *p* < 0.05

**Table 12:** Regression of board of director attributes and real activity manipulation

Dependent Variable	(13) Pre-Period (2015&2016)		(14) Post-Period (2018&2019)		(15) Full-Period		(16) Pre-Period (2015&2016)		(17) Post-Period (2018&2019)		(18) Full-Period	
	DIS_ERM		DIS_ERM		DIS_ERM		P_RM		P_RM		P_RM	
Independent Variables	Coefficient	t-value	Coefficient	t-value	Coefficient	t-value	Coefficient	t-value	Coefficient	t-value	Coefficient	t-value
BIND	−1.094	(−1.42)	−0.668	(−1.25)	−0.046	(−0.15)	−0.416^**^	(−1.90)	0.224	(1.18)	−0.026	(−0.30)
BEXP	−0.618	(−1.25)	−0.170	(−0.37)	−0.374	(−1.56)	−0.116	(−0.65)	0.062	(0.38)	−0.044	(−0.61)
CEOD	−0.084	(−0.41)	0.000	(.)	−0.016	(−0.13)	−0.026	(−0.27)	0.000	(.)	−0.060	(−1.43)
BDIV	0.078	(0.12)	0.946^**^	(1.74)	−0.250	(−0.96)	0.152	(0.85)	−0.041	(−0.21)	0.106	(1.41)
FD	−0.167	(−0.27)	−1.666^***^	(−2.73)	0.025	(0.09)	0.185	(0.78)	−0.071	(−0.33)	0.197^***^	(2.28)
BMEET	0.028	(1.19)	0.012	(0.57)	0.018	(1.51)	−0.006	(−0.81)	0.000	(0.06)	0.002	(0.73)
BSIZE	0.026	(0.33)	−0.042	(−0.58)	−0.011	(−0.30)	0.005	(0.17)	0.000	(0.00)	−0.008	(−0.78)
ACIND	0.403	(1.22)	−0.003	(−0.01)	0.233	(1.32)	0.030	(0.26)	−0.022	(−0.13)	−0.047	(−0.85)
ACEXP	−0.042	(−0.16)	−0.060	(−0.26)	0.081	(0.63)	−0.109	(−0.85)	−0.002	(−0.03)	0.024	(0.59)
BIG4	0.263^**^	(1.71)	−0.034	(−0.14)	0.235^***^	(2.55)	−0.052	(−0.65)	0.035	(0.42)	0.020	(0.66)
ROA	−0.006	(−0.77)	−0.003	(−0.45)	−0.006	(−1.63)	−0.003	(−1.13)	−0.010^***^	(−4.20)	−0.010^***^	(−10.00)
LEVE	0.204	(0.55)	−0.057	(−0.16)	−0.173	(−1.02)	0.120	(0.88)	−0.080	(−0.63)	−0.115^***^	(−2.25)
FAGE	−0.044	(−1.36)	0.043	(1.15)	0.007	(0.67)	−0.017	(−1.37)	0.030^***^	(2.20)	−0.014^***^	(−4.15)
Tangibility	−0.122	(−0.30)	−0.310	(−0.85)	−0.314^**^	(−1.88)	−0.036	(−0.30)	0.142	(1.08)	0.004	(0.08)
FSIZE	−0.062	(−0.33)	0.055	(0.32)	−0.347^***^	(−5.64)	−0.096	(−1.50)	−0.244^***^	(−3.95)	−0.063^***^	(−3.37)
LOSS(D)	−0.120	(−1.21)	−0.025	(−0.30)	0.007	(0.15)	0.004	(0.15)	−0.003	(−0.11)	0.003	(0.23)
CFO	0.004^***^	(5.97)	−0.003^***^	(−2.09)	0.006^***^	(10.52)	−0.056	(−0.73)	−0.000	(−0.17)	−0.001^***^	(−2.43)
SALES	−0.045	(−0.50)	0.063	(0.53)	−0.091^**^	(−1.83)	−0.230^***^	(−7.00)	−0.251^***^	(−5.91)	−0.160^***^	(−9.69)
INV	−0.334	(−0.56)	0.142	(0.30)	−0.404	(−1.57)	1.303^***^	(6.24)	1.402^***^	(8.40)	0.898^***^	(12.20)
Constant	2.265	(1.21)	−1.128	(−0.59)	3.222^***^	(5.38)	1.616^***^	(2.32)	0.931	(1.37)	1.247^***^	(6.70)
N	578		398		1179		384		397		980	
Groups (Firms)	212		209		223		203		209		218	
*R*^2^	0.129		0.090		0.166		0.521		0.520		0.418	
F-Statistics	2.704**		0.935		9.833***		9.335***		10.236***		28.091***	

*t* statistics in parentheses, ^**^ *p* < 0.10, ^***^ *p* < 0.05

The possible factors that will be responsible for this will be the ownership concentration and family businesses in Pakistan, which do not allow female directors to act an independent way and in the best interests of all shareholders. Furthermore, the family female director is common in the Pakistani boardroom, which always went with the majority family member’s decision. This finding is consistent with [50, 68]. Although the outcomes of this research contradict the study by [72] Pakistani context, which posited that a gender-diverse board reduces EM in PSX non-financial listed firms, they further added that female presence in the board improves and strengthens the effectiveness of the board, which is then useful for curtailing EM.

### Additional analysis

In addition to the main results, in this research study we interacted the dummies of the pre- and post-period of CCG-2017 with the revised BOD features to check the validity of the main regression as shown in Table 10. The results of Table 10 show that the interaction terms of BIND*POST_17 and BEXP*POST_2017 are negatively related to MCDA, the proxy of EM. Furthermore, their relationship is statistically significant not only in the post-period of CCG-2017 but also in full-period regression (2015 to 2019).

Moreover, in the BOD attributes and the interaction term of the pre-CCG-2017 dummy, as shown in regression (4) reported that BODs features are not significantly related to EM, which is similar to the regression results of Table 10.

In Tables 13 and 14, we did additional analysis in order to check whether the revised BOD attributes in CCG-2017 have any impact on the EM in large and small firms as well as in family-owned and non-family-owned firms. As these findings indicate that board independence and expertise have an impact on mitigating EM in PSX-listed non-financial firms, the question of whether these findings apply to small- vs. large-sized firms, as well as family-owned and non-family-owned firms, arises. Therefore, we have conducted additional analysis towards this end. Table 13 shows the results of small and large firms, which were divided by firm size. The findings show that in regression 20, the board independence is effectively curbing the EM practices in the post-CCG-2017 period, while in the pre-period and full-period regression, none of the BOD variables is related to EM estimated under [48] model. Furthermore, in the small-sized firms, board independence and expertise are negatively related to EM in all the regressions from 22 to 24. According to these findings, only board independence and expertise are negatively related to EM, whereas board female directorship and CEO duality are not.

**Table 13:** Regression results of board of directors attributes and earnings manipulation of small vs large firms

Dependent variable	Large firms (Firm size)								Small firms (Firm size)					
	(19) Pre-Period (2015&2016)				(20) Post-Period (2018&2019)		(21) Full-Period		(22) Pre-Period (2015&2016)		(23) Post-Period (2018&2019)		(24) Full-Period	
	MCDA				MCDA		MCDA		MCDA		MCDA		MCDA	
Independent variables		Coefficient		t-value	Coefficient	t-value	Coefficient	t-value	Coefficient	t-value	Coefficient	t-value	Coefficient	t-value
BIND		−0.164^***^		(−2.11)	−0.301^***^	(−2.22)	−0.153	(−1.64)	−0.312^***^	(−2.55)	−0.309^***^	(−2.53)	−0.264^**^	(−1.96)
BEXP		0.108^**^		(1.80)	−0.121	(−1.18)	0.090	(1.31)	−0.259^***^	(−2.52)	−0.212^***^	(−1.99)	−0.294^***^	(−2.45)
BDIV(%)		−0.005		(−0.07)	0.129	(1.02)	−0.007	(−0.08)	0.070	(0.70)	0.148	(1.45)	0.053	(0.51)
CEOD		0.066		(1.60)	−0.007	(−0.07)	0.056	(0.91)	0.005	(0.11)	0.052	(0.96)	0.018	(0.32)
FD		−0.237^***^		(−2.61)	0.002	(0.01)	−0.186	(−1.59)	−0.027	(−0.26)	−0.170	(−1.51)	−0.074	(−0.69)
BMEET		0.003		(0.92)	−0.001	(−0.24)	0.001	(0.25)	0.003	(0.76)	0.002	(0.46)	0.004	(0.95)
BSIZE		−0.007		(−0.78)	0.014	(0.74)	−0.007	(−0.59)	0.030^***^	(2.12)	0.012	(0.88)	0.014	(0.87)
ACIND		0.081		(1.47)	0.256^***^	(2.50)	0.120^**^	(1.71)	0.116^**^	(1.80)	0.150^***^	(2.05)	0.127^**^	(1.87)
ACEXP		−0.039		(−1.03)	−0.049	(−0.76)	−0.053	(−1.13)	0.107^***^	(2.00)	0.049	(0.91)	0.111^**^	(1.94)
BIG4		−0.012		(−0.46)	0.033	(0.67)	−0.001	(−0.02)	0.018	(0.40)	0.027	(0.59)	0.026	(0.48)
ROA		0.004^***^		(4.55)	−0.002	(−1.34)	0.005^***^	(4.20)	0.001	(0.48)	0.000	(0.18)	0.000	(0.10)
LEVE		−0.010		(−0.21)	−0.094	(−1.09)	0.004	(0.07)	0.016	(0.25)	0.074	(1.13)	0.038	(0.57)
FAGE		0.000		(0.08)	0.017^***^	(2.85)	0.003	(0.70)	0.013^***^	(2.68)	0.005	(0.89)	0.012^***^	(2.45)
Tangibility		0.050		(1.01)	0.155^**^	(1.73)	0.064	(1.00)	−0.046	(−0.83)	−0.071	(−1.18)	−0.076	(−1.32)
FSIZE		0.037^***^		(2.27)	0.009	(0.26)	0.023	(0.93)	0.024	(0.80)	0.065^***^	(2.66)	0.020	(0.62)
LOSS(D)		0.015		(1.11)	0.044^**^	(1.82)	0.014	(0.78)	0.054^***^	(3.19)	0.053^***^	(3.01)	0.056^***^	(3.19)
CFO		−0.720^***^		(−28.31)	−0.001	(−1.42)	−0.710^***^	(−24.41)	−0.002^***^	(−5.88)	−0.001^***^	(−4.21)	−0.002^***^	(−5.91)
SALES		0.027^**^		(1.83)	0.024	(0.90)	0.000	(0.03)	0.003	(0.16)	0.047^***^	(2.07)	0.018	(0.75)
INV		−0.139^**^		(−1.94)	0.530^***^	(4.58)	−0.176^***^	(−2.00)	0.520^***^	(5.15)	0.555^***^	(5.44)	0.505^***^	(4.87)
Constant		−0.269		(−1.57)	−0.972^***^	(−2.81)	−0.258	(−1.05)	−0.980^***^	(−3.59)	−0.883^***^	(−3.66)	−0.781^***^	(−2.84)
N		719			673		512		650		684		464	
Groups (Firms)		209			212		128		214		210		122	
*R*^2^		0.40			0.007		0.43		0.27		0.24		0.32	
F-Statistics		54.17***			3.48***		39.11***		8.38***		7.68***		8.25***	

*t* statistics in parentheses, ^**^ *p* < 0.10, ^***^ *p* < 0.05

**Table 14:** Regression results of board of directors attributes and earnings manipulation of family and non-family firms

Dependent Variable	Family firms						Non-family firms					
	(25) Pre-Period (2015&2016)		(26) Post-Period (2018&2019)		(27) Full-Period		(28) Pre-Period (2015&2016)		(29) Post-Period (2018&2019)		(30) Full-Period	
	MCDA		MCDA		MCDA		MCDA		MCDA		MCDA	
Independent Variables	Coefficient	t-value	Coefficient	t-value	Coefficient	t-value	Coefficient	t-value	Coefficient	t-value	Coefficient	t-value
BIND	−0.066	(−1.40)	0.017	(0.24)	−0.062	(−1.21)	−0.005	(−0.07)	−0.005	(−0.06)	0.061	(0.52)
BEXP	−0.017	(−0.56)	−0.022	(−0.48)	−0.022	(−0.63)	−0.020	(−0.48)	−0.090^***^	(−1.97)	−0.086	(−1.41)
BDIV (%)	0.039	(1.39)	0.065	(1.56)	0.022	(0.62)	0.056	(1.58)	0.043	(1.11)	0.046	(0.92)
CEOD	0.015	(0.80)	−0.004	(−0.12)	0.006	(0.29)	−0.019	(−0.82)	−0.014	(−0.57)	−0.004	(−0.12)
FD	−0.010	(−0.59)	−0.009	(−0.37)	−0.039^**^	(−1.78)	−0.001	(−0.27)	−0.002	(−0.76)	−0.002	(−0.76)
BMEET	0.000	(0.06)	0.000	(0.18)	−0.000	(−0.17)	0.007	(1.59)	−0.002	(−0.33)	0.007	(0.96)
BSIZE	−0.001	(−0.41)	0.001	(0.16)	0.000	(0.01)	0.087^**^	(1.94)	0.131^***^	(2.43)	0.084	(1.22)
ACIND	0.048	(1.54)	0.065	(1.29)	0.010	(0.28)	−0.012	(−0.48)	0.022	(0.82)	0.009	(0.23)
ACEXP	0.018	(0.99)	−0.002	(−0.09)	0.020	(0.95)	−0.014	(−1.22)	−0.014	(−1.09)	−0.031	(−1.54)
BIG4	0.003	(0.40)	−0.001	(−0.13)	0.009	(0.97)	−0.002^***^	(−2.46)	−0.001	(−1.63)	−0.002^***^	(−2.03)
ROA	0.005^***^	(9.06)	−0.002^***^	(−2.50)	0.005^***^	(7.30)	−0.030	(−1.21)	−0.050^**^	(−1.87)	−0.042	(−1.21)
LEVE	0.007	(0.36)	−0.049^**^	(−1.77)	0.037	(1.58)	0.000	(0.02)	−0.000	(−0.26)	−0.000	(−0.15)
FAGE	−0.000	(−0.05)	0.000	(0.72)	0.000	(0.70)	−0.030	(−1.23)	−0.053^***^	(−2.07)	−0.048	(−1.31)
Tangibility	0.063^***^	(3.52)	−0.045^**^	(−1.86)	0.056^***^	(2.73)	0.006	(1.39)	0.013^***^	(2.79)	0.016^***^	(2.40)
FSIZE	0.001	(0.31)	0.008^**^	(1.93)	0.002	(0.67)	0.009	(0.63)	0.022	(1.38)	0.018	(0.82)
LOSS(D)	0.018^***^	(1.99)	0.023	(1.60)	0.015	(1.49)	0.014	(0.48)	0.041	(0.95)	0.072	(1.12)
CFO	−0.634^***^	(−31.70)	−0.002^***^	(−7.90)	−0.630^***^	(−29.87)	−0.003^***^	(−8.81)	−0.003^***^	(−8.28)	−0.003^***^	(−7.69)
SALES	0.005	(0.91)	0.000	(0.02)	0.013^***^	(2.07)	−0.025^***^	(−3.25)	0.012	(1.38)	−0.013	(−1.07)
INV	0.025	(0.80)	0.200^***^	(4.42)	0.009	(0.26)	0.168^***^	(3.59)	0.092^**^	(1.96)	0.183^***^	(2.58)
Constant	−0.050	(−1.32)	−0.081	(−1.50)	−0.070	(−1.53)	−0.089^**^	(−1.71)	−0.065	(−1.19)	−0.141^**^	(−1.78)
N	759		757		615		608		597		357	
Groups (Firms)	211		212		134		211		210		82	
*R*^2^	0.56		0.14		0.59		0.18		0.16		0.22	
F-Statistics	1099***		120***		984***		136***		114***		98.23***	

*t* statistics in parentheses, ^**^p < 0.10, ^***^p < 0.05

Furthermore, in Table 14, the revised BOD attributes in CCG-2017 are also checked with EM estimated under the [48] model in the family and non-family firms. In regressions 25 to 27, the results of family firms are given. According to the results of family firms, CCG-2017 revised BOD attributes had no effect on reducing EM. Moreover, in family firms in PSX, the BOD attributes are not related to EM not only in the post-CCG-2017 period but also in the pre- and full-period regression. The only board attribute which is negatively related to EM in the full period regression is the family director (FD), which has a negative and significant relationship with EM in the full period regression, which is according to socioemotional wealth theory. Additionally, when the revised BOD attributes in CCG-2017 are linked with non-family firms in the regressions 28 to 30, the results show that none of the BOD features are significantly related to EM, except for board expertise, which is negatively related to EM in the post-CCG-2017 period. The results of Table 14 show that among the non-family firms, only financially expert directors in the board effectively counter the management practices of EM, while other BOD attributes such as independence, female representation, and CEO duality are not related to EM. Lastly, for the brevity of this research study, additional analysis has not been carried out with RAM proxies.

## Conclusion

Although the BOD features and EM literature are extensive, very few and limited works are available in the related literature focusing on the Pakistani family-oriented and concentrated context. Furthermore, previous related studies focused on BODs features and EM, whereas this research study focuses on the aforementioned relationship with country-specific CCG-2017 regulation, which is currently lacking in the literature. Following the identified research gap, this research study takes into consideration the CCG-2017 regulation relating to the board attributes, i.e., independence, financially expert directors, CEO Duality and female directors. These revised BOD attributes in the CCG-2017 are regressed on EM to check whether there is any influence of the said revision on EM before and after the CCG-2017 of the non-financial listed firms of PSX. The sample included 323 firms over a 5-year period, with 2 years pre- and post-period of the CCG-2017 from 2015 to 2019. The findings of this study show that, similar to other studies, independent and financially expert directors are instrumental after the implementation of CCG-2017 in limiting earnings manipulation (discretionary accruals). Although board independence and expertise are vital in constraining discretionary accruals, they have no role in REM. In addition to this, contrary to the study’s hypotheses, female directors are positively associated with not only accrual manipulation but also abnormal cash flow manipulation. The possible reason for the findings may be the tokenism hypothesis, which states that the inclusion of female directors is just for compliance, nothing more than that, and to avoid any disciplinary action. Furthermore, another possible reason may be that there are family female directors on the board who have less decision-making power as compared to family-dominated male directors. Additionally, Pakistan has family-oriented and concentrated ownership where female directors are not included in the board due to their qualities rather than due to nepotism and friendly ties with family businesses. The study’s results contribute to the corporate governance and EM literature by focusing on country-specific regulation on BOD features.

### Implications of the study

This research study has implications for the regulator, policy makers, and managers/owners of listed firms. The implementation of CCG-2017 was a much-needed step for better monitoring and safeguarding the minority shareholders' rights. This research study provides useful insights to potential investors, analysts and various types of fund managers to focus on those firms for their investments that have independent, diverse and expert directors, as they will reduce EM practises and provide more transparency in financial reporting. The regulator needs to augment the authentic independence of independent/outside directors in listed firms (concentrated ownership context) of Pakistan. In addition to this, the inclusion of a female directorship was a much needed step, but it needs to be converted into reality and must not be considered as a fill in the blanks for meeting the guidelines rather than be considered a new direction for better board monitoring and diverse opinions in the boardrooms. Both regulators and management of the firms need to cultivate gender diversity on the boards. Lastly, the revised clauses of BOD in the CCG-2017 should be followed in true spirit, and the regulator (SECP) should ensure that those firms that violate the said rules are taken into account.

### Limitations of the study

Like other studies, this paper also has limitations that may provide avenues for future researchers to pursue. Firstly, since the sample size of this study consists of non-financial listed firms, its findings cannot be generalized to financial listed firms on PSX. Secondly, this research study included non-financial listed firms of PSX. However, there are non-listed firms in Pakistan, which future researchers may consider. Thirdly, due to data constraints, this study took two years before and after CCG-2017, but future researchers may extend it to three years. Lastly, this study has linked BOD-related variables with EM by estimating EM via [48, 58] models. The findings with these variables may be limited in validity, which may be associated with the EM estimation model. Therefore, future researchers may consider other proxies for EM estimations, both accrual-based proxies as well as REM proxies. Moreover, future research may link CCG-2017 with firm performance, CSR, and agency costs, etc., by incorporating more explanatory variables. Moreover, future studies may include the COVID-19 pandemic via linking BOD with EM by following the studies of [69, 70].

## Data Availability

We have used secondary sources to complete our study. No new data are used or produced in this study.

## Appendix

See Table 15.

**Table 15:** Variable definitions

Variables	Description	
*Dependent variables*		
MCDA	Discretionary accruals calculated under McNichols, [48] model	
REALEM	Aggregate Real activity Manipulation by adding abnormal production cost with abnormal cash flow form operation and abnormal discretionary expenses [49]	
DIS ERM	abnormal discretionary expenses	Abnormal discretionary expenses estimated using [58] model
RCFO	abnormal cash flow form operation	Abnormal cash flow form operation estimated using [58] model
PRO RM	abnormal production cost	Abnormal production cost estimated using [58] model
*Board of directors variables*		
BIND	Board Independence	Independent directors/ Size of the board
BEXP	Board Expertise	Those directors who have accounting and finance related education/experience by size of the board
BDIV	Board Female directorship	Female directors/Size of the board
FD	Family directors	Family directors/Size of the Board
BMEET	Board meetings	The number of the board meetings in a year
BSIZE	Board Size	The number of directors in the board
CEOD	CEO Duality	Dummy variable taking 1 if CEO’s holding the position of chairman 0 otherwise
*Control variables*		
ACIND	Audit Committee Independence	Number of directors in audit committee/ Size of the Audit Committee
ACEXP	Audit Committee Expertise	Those directors who have accounting and finance related education/experience by size of the audit committee
BIG4	BIG 4 Audit Firms	A firm audited by Big 4 audit firm will take 1 and others will take 0
ROA	Return on Assets	Earnings before tax/Total Assets[18]
LEVE	Leverage	Short term& long term Debt/Total Assets
FAGE	Firm Age	The age of the firm since its incorporation
TANG	Tangibility	Fixed Assets/ Total Assets
FSIZE	Firm Size	Natural log of Total Assets[62]
LOSS(D)	Loss dummy	A Dummy variable which is taking the value of 1 if the firm is reporting loss in the annual report other wise 0
CFO	Cash flow from operation	Cash flow from operation/Total Assets
SALES	Sales/Total assets	Revenues/Total Assets
INV	Inventory/total assets	Inventory at the end of the period/Total Assets

## Abbreviations

BOD: Board of directors
CG: Corporate governance
CCG: Corporate governance code
SECP: Securities and Exchange Commission of Pakistan
HEC: Higher Education Commission
CEO: Chief Executive Officer
SBP: State Bank of Pakistan
PSX: Pakistan stock exchange
EM: Earnings management
SOX: Sarbanes–Oxley Act

## Footnotes

1 https://www.psx.com.pk/psx/themes/psx/documents/legal-framework/SECP/regulations/listed-companies-(ccg)-regu-2017/ListdCmpCodeOfCorpGovern017.pdf

2 https://www.secp.gov.pk/laws/regulations/.
